# Isometric Exercise Training for Managing Vascular Risk Factors in Mild Cognitive Impairment and Alzheimer’s Disease

**DOI:** 10.3389/fnagi.2017.00048

**Published:** 2017-03-03

**Authors:** Nicole C. L. Hess, Neil A. Smart

**Affiliations:** School of Science and Technology, University of New EnglandArmidale, NSW, Australia

**Keywords:** Alzheimers disease, mild cognitive impairment, vascular risk factors, hypertension, blood pressure, reactive hyperemia, isometric exercise training

## Abstract

Alzheimer’s disease (AD) is the most common form of dementia diagnosed amongst the elderly. Mild cognitive impairment (MCI) is a condition often indicative of the earliest symptomatology of AD with 10%–15% of MCI patients reportedly progressing to a diagnosis of AD. Individuals with a history of vascular risk factors (VRF’s) are considered high risk candidates for developing cognitive impairment in later life. Evidence suggests that vascular injury resulting from untreated VRF’s promotes progression from MCI to AD and exacerbates the severity of dementia in AD, and neuroimaging studies have found that the neurodegenerative processes associated with AD are heavily driven by VRF’s that promote cerebral hypoperfusion. Subsequently, common links between vascular disorders such as hypertension and neurodegenerative disorders such as AD include compromised vasculature, cerebral hypoperfusion and chronic low grade inflammation (a hallmark of both hypertension and AD). Exercise has been demonstrated to be an effective intervention for blood pressure management, chronic low grade inflammation and improvements in cognition. Data from recent analyses suggests that isometric exercise training (IET) may improve vascular integrity and elicit blood pressure reductions in hypertensives greater than those seen with dynamic aerobic and resistance exercise. IET may also play an effective role in the management of VRF’s at the MCI stage of AD and may prove to be a significant strategy in the prevention, attenuation or delay of progression to AD. A plausible hypothesis is that the reactive hyperemia stimulated by IET initiates a cascade of vascular, neurotrophic and neuro-endocrine events that lead to improvements in cognitive function.

## Introduction

**Figure 1:**
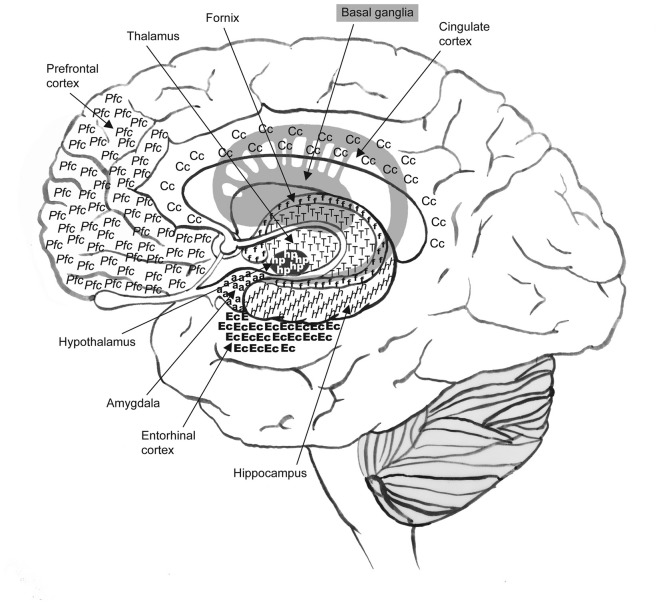
**Alzheimer’s disease (AD) is responsible for severe cortical atrophy in selective regions of the brain such as the frontal and prefrontal cortex, and the temporal, medial-temporal and limbic areas.** Neural structures associated with these regions include the thalamus, basal ganglia, cingulate cortex, fornix, hypothalamus, amygdala, hippocampus and entorhinal cortex. One of the earliest indicators of AD, identified by imaging studies, is the presence of cerebral hypoperfusion in temporoparietal regions such as the entorhinal and hippocampal areas.

Alzheimer’s disease (AD) is a progressive neurodegenerative dementing disorder responsible for severe cortical atrophy in selective regions of the brain such as the temporal, medial-temporal, limbic, frontal and prefrontal cortices (Braak et al., 2006; Querfurth and LaFerla, 2010; Archer et al., 2011), see Figure 1. The decay of these neural structures is deleterious to a number of cognitive and functional domains including learning, memory, attention, motivation, executive function, motor function and global cognition (Archer, 2011; Archer et al., 2011). Mild cognitive impairment (MCI), often considered to be the earliest symptomatic manifestation of AD (DeCarli, 2003; Etgen et al., 2011) is also accompanied by significant, non-normative atrophy of the medial temporal and temporal cortices (Braak et al., 2006; Archer et al., 2011). Currently, there is no cure or effective treatment for AD and despite decades of investigation the pathogenesis of sporadic (late-onset) AD remains both elusive and controversial. Knowledge of the disease pathogenesis would likely aid in the development of an effective treatment. The Amyloid cascade hypothesis initially suggested by Glenner and Wong (1984) still remains somewhat of an axiom. This hypothesis purports genetic causation and proposes that the Aβ peptide initiates a cascade of events that manifest in amyloid plaque deposition and the hyperphosphorlation of tau protein, forming neurofibrillary tangles. The end result of these events is neuronal injury and loss, and ultimately, the development of AD. This theory has been criticized for its inability to explain the etiology of these hallmark pathologies and also for its inability to deliver an effective treatment (de la Torre, 2002). Current pharmacotherapy does not act on these indicators and has minimal effect on the symptomatic presentations of the disease (de la Torre, 2002, 2004; Birks, 2006; Campbell et al., 2013). Consequently, investigators continue to examine alternative hypotheses to explain AD pathogenesis. Over the past two decades vascular hypotheses of AD have received considerable attention (de la Torre and Mussivand, 1993; Zlokovic, 2011); these theories focus on a non-amyloidogenic pathway of AD that is driven by vascular risk factors (VRF’s) such as hypertension, atherosclerosis, hyperlipidemia and cerebrovascular disease which may ultimately lead to cerebral hypoperfusion and as a consequence result in neuronal dysfunction leading to cognitive decline and AD. There is convincing research to support a vascular hypothesis of AD; the severity of dementia in AD patients has been found to be exacerbated by the presence of cerebral ischemeic lesions (Iadecola, 2010), neuroimaging studies have identified that damaged and dysfunctional cerebral microcirculation is one of the earliest predictors of AD (Hirao et al., 2005), hypertension is reported to cause injury to the vascular system (Brickman et al., 2010) and is associated with cerebral vascular pathology, hypoperfusion and cognitive decline (Brown and Thore, 2011). Exercise has long been recommended and demonstrated as an effective therapeutic intervention for hypertension (Cardoso et al., 2010; Cornelissen and Smart, 2013), MCI and AD and has been associated with marginal improvements in cognitive performance outcomes (Colcombe and Kramer, 2003; Heyn et al., 2004; van Uffelen et al., 2008; Aarsland et al., 2010; Baker et al., 2010; Smith et al., 2010; Sofi et al., 2011; Hess et al., 2014). Most studies have focused on aerobic exercise due to shear wall stress and the subsequent release of nitric oxide (NO; Keller et al., 2001; Venturelli et al., 2011), however, an increasing body of evidence supports the role of isometric exercise training (IET) to affect significant reductions in resting systolic and diastolic blood pressures (DBP) in both hypertensive and normotensive men and women (Devereux et al., 2010; Wiles et al., 2010; Badrov et al., 2013a,b; Millar et al., 2013). Unlike aerobic exercise, the potential for IET to assist with improvements in cognitive performance have not yet been investigated. The physiological mechanisms elicited by IET are not fully understood and are still under investigation; however, it may be the case that resulting from increased blood flow due to small repeated bouts of ischemia, IET elicits increases in angiogenesis, neuro-endocrine function and metabolites such as beta endorphins and prostaglandins (Stiller-Moldovan et al., 2012; Wong and Wright, 2014; Wong et al., 2015). Subsequently, it may be the case that in conjunction with its anti-hypertensive effects, isometric exercise may also offer the potential to elicit improvements in cognitive performance.

The following article reviews; subsets of cognitive impairment; the clinical and neuropathological differences between AD and vascular dementia (VaD); Vascular hypotheses of AD and the contribution of vascular pathology to cognitive decline in AD; purported linkages between VRF’s and cognitive impairment, with a specific focus on hypertension; and the shared pathological events prevalent in hypertension and AD. We then discuss exercise as a modifiable risk factor for hypertension and cognitive decline. We further consider the potential benefits and efficacy of remote limb ischemia and IET as non-pharmacological therapies for preventing and/or attenuating the progression of MCI to incidence of sporadic AD and as interventions that might assist those already diagnosed with early stage sporadic AD.

## Mild Cognitive Impairment Due to Alzheimers Disease

Cognitive impairment without dementia, such as age-associated cognitive decline (AACD) and age-associated memory impairment (AAMI), is considered to fall within the normative realms of brain aging (Ritchie et al., 2001; Alzheimers Association, 2012). Whereas MCI is a condition that is characterized by a deterioration in cognitive abilities and memory beyond that expected for a person’s age and level of education, but without notable loss of global cognition or activities of daily living (Duara et al., 2010; Albert et al., 2011; Archer et al., 2011; Etgen et al., 2011; Alzheimers Association, 2012). Depending on the cognitive functions that are affected, MCI is further classified into either nonamnestic MCI or amnestic MCI (aMCI; Alzheimers Association, 2012). Nonamnestic MCI affects cognitive domains other than memory such as the sequencing of complex tasks, judgment and decision making skills, and visual perception, whereas aMCI primarily affects memory and is more likely to progress to AD (Archer et al., 2011; Etgen et al., 2011; Alzheimers Association, 2012).

While not all cases of MCI will progress to a clinical diagnosis of AD or dementia (DeCarli, 2003; Panza et al., 2005) within the space of 12 months, 10%–15% of MCI cases reportedly will convert to AD (DeCarli, 2003; Petersen et al., 2009; Etgen et al., 2011). However, these conversion rates have been shown to vary significantly among studies and clinical populations based on differences in diagnostic criteria, sampling populations and assessment protocols (Panza et al., 2005). Despite these variances, MCI is considered to represent the earliest symptomatic indications of AD (DeCarli, 2003; Helzner et al., 2009; Etgen et al., 2011; Li et al., 2011) and is viewed as a prodromal, pathological condition rather than as a consequence of the normative aging process (Petersen, 2004; Albert et al., 2011).

## Alzheimers Disease and Vascular Dementia

AD and VaD are the two most common forms of dementia diagnosed amongst older adults with AD being most common (Qiu et al., 2005). The severity and extent of AD-related neurodegenerative atrophy grows as a function of time selectively and predictably destroying memory functions, cognitive performance and functional abilities at each stage. Whereas, in VaD the presentation of dementia is attributed to dysfunctional vascular mechanisms (Graham et al., 2004) and is not accompanied by the hallmark neurodegenerative processes prevalent in AD. Pathological lesions associated with VaD include, ischemic or hemeorrhagic infarct(s); atherosclerosis (basal, peripheral or meningeal); microvascular changes; parenchymal changes in cortex, white matter, basal ganglia, brain stem and cerebellum; hippocampal sclerosis; perivascular and parenchymal gliosis (Kalaria, 2016). Throughout the progression of VaD memory remains intact with attentional and executive functioning disproportionately impaired (Graham et al., 2004; Agüero-Torres et al., 2006).

**Figure 2:**
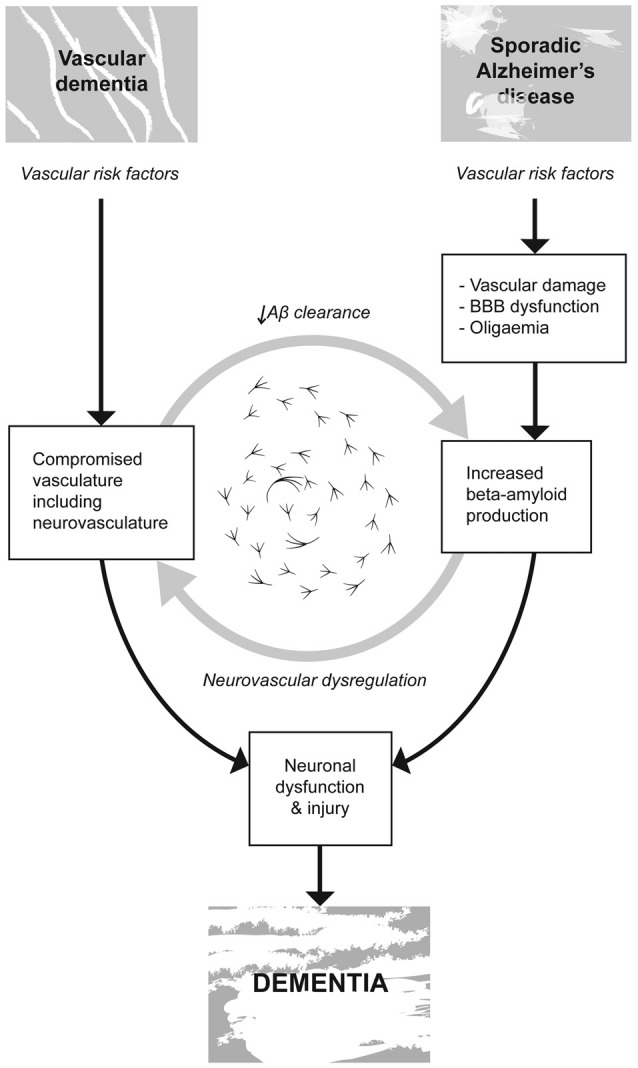
**Vascular risk factors (VRF’s) such as hypertension, atherosclerosis, hyperlipidemia and cerebrovascular disease are linked to cognitive disorders such as vascular dementia (VaD) and sporadic AD.** Vascular damage associated with aging, hypertension and other VRF’s is thought to; inhibit both the delivery of nutrients to the brain and the clearance of toxic metabolites, compromise the integrity of the blood brain barrier (BBB) promoting the accumulation and propagation of the hallmark proteogenic pathologies associated with AD. VaD is not accompanied by these same hallmark neurodegenerative processes. In both VaD and AD, the homeostatic disruption of altered cerebral vasculature is believed to promote cellular disruption, cell death and cognitive impairment ultimately resulting in dementia.

Traditionally these two diseases have been studied separately, however, increasingly, over the past two decades investigators have been able to link the contribution of VRF’s such as hypertension, atherosclerosis, hyperlipidemia and cerebrovascular disease to cognitive disorders such as MCI and sporadic AD (Viswanathan et al., 2009; de la Torre, 2010; Laukka et al., 2010; Choi, 2012). Vascular damage associated with aging, hypertension and other VRF’s is thought to inhibit both the delivery of nutrients to the brain and the clearance of toxic metabolites. The ensuing homeostatic disruption of altered cerebral vasculature is purported to promote cellular disruption, cell death and cognitive impairment (Iadecola et al., 2009), see Figure 2.

## The Vascular Hypothesis of Alzheimers Disease

The vascular hypothesis of AD proposes that cerebral hypoperfusion is the causal factor in disease development (de la Torre and Mussivand, 1993). The hypothesis recognizes an intimate link between vascular dysfunction and neuronal dysfunction and highlights the importance of the circulatory system to brain functions. The hypothesis proposes that sporadic AD is a multifactorial disease fueled by VRF’s such as hypertension, atherosclerosis, cardiac disease, stroke and diabetes, that contribute to chronic brain hypoperfusion/oligemia (reduced cerebral blood flow). Obstructed cerebral blood flow prevents the efficient delivery of nutrients such as oxygen, glucose and micronutrients to the brain and compromises energy metabolism and neural activity. Similar to the vascular hypothesis, the two-hit hypothesis also proposes that a non-amyloidogenic pathway driven by VRF’s and reduced cerebral perfusion might be contributing to the development of late onset AD (Zlokovic, 2011).

The two-hit theory proposes that cerebral hypoperfusion and an over accumulation of the Aβ peptide triggers the hyperphosphorylation of p-tau which manifests in neurofibrillary tangles, neuronal degeneration and eventually AD (Zlokovic, 2011). This theory suggests that VRF’s play a pivotal role in the pathogenesis of the disease. Hit one proposes that vascular damage compromises the integrity of the blood brain barrier (BBB) and facilitates a reduction in cerebral blood flow. Vascular injury inhibits the clearance of Aβ at the BBB; this in turn mediates increased production of Aβ and results in the over accumulation of neurotoxic levels of this peptide. Both the aggregation of toxic levels of Aβ, and cerebral hypoperfusion promote early neuronal dysfunction. Hit two proposes that continuing increases in Aβ accumulation exacerbates neuronal dysfunction, is a catalyst for neurodegeneration and AD, and promotes self-propagation of the disease.

## Vascular Pathology and Cognitive Decline in AD

Pathological changes to the cerebral microvasculature precede and/or accompany vascular disorders such as hypertension, neurovascular disorders such as AD and cognitive decline (Brown and Thore, 2011). Individuals with a history of VRF’s and vascular disease are considered high risk candidates for developing cognitive impairment in later life (Korf et al., 2004; Solfrizzi et al., 2004; Laukka et al., 2010). Evidence suggests that vascular injury exacerbates the severity of dementia in AD and that the neurodegenerative process is heavily driven by vascular factors (Heyman et al., 1998; Vermeer et al., 2003; Song et al., 2007; Schneider et al., 2009; White, 2009). Additionally, vascular lesions and VRF’s have been reported to increase the rate of cognitive decline and accelerate the disease progression (Helzner et al., 2009).

Imaging studies have identified cerebral hypoperfusion in selective neural regions as one of the earliest indicators of AD, specifically in the temporoparietal regions such as the entorhinal, transentorhinal and hippocampal areas (areas linked to memory function, and the first regions to be afflicted with AD neuropathology), see Figure 1 (de la Torre, 2004; Hirao et al., 2005; Ruitenberg et al., 2005). In these studies, individuals displaying hypoperfusion and complaining of memory problems or diagnosed with MCI went on to develop AD (de la Torre, 2004; Hirao et al., 2005), whereas, those individuals who showed normal cerebral blood flow did not convert to AD during the observation period. Other studies have identified that cerebral hypoperfusion accompanies hippocampal atrophy (Jack et al., 1999; Ruitenberg et al., 2005).

Neuroimaging research suggests that the clinical symptoms associated with sporadic AD result from neurodegeneration not amyloid deposition and that cognitive decline is directly related to the neurodegenerative process of the disease pathology and not amyloidosis (Pedersen et al., 2003; Hiscock et al., 2004; Jack et al., 2009). These studies also suggest that there is no association between the rate of neurodegeneration and the rate of amyloid deposition. Consequently, if the neurodegenerative processes of AD are intensified by vascular factors then timely interventions that address these risk factors and reinstate the delivery of a nutrient rich oxygenated blood supply to the brain may ameliorate or attenuate the neurodegenerative process and disease trajectory.

## Hypertension

Hypertension is associated with cerebrovascular pathology, hypoperfusion (Brown and Thore, 2011) and cognitive decline (Reitz et al., 2007). MRI studies have demonstrated a link between brain atrophy and untreated hypertension (Launer et al., 1995) and results from the “The Honolulu Asia Aging Study” (HAAS; Korf et al., 2004) show that hippocampal atrophy can be linked to untreated hypertension in midlife, and that a positive correlation exists among systolic blood pressure (SBP), DBP and burden of neural AD pathology. Hypertension causes injury to the vascular system and is associated with an elevated burden of neural white matter lesions (Brickman et al., 2010). Hypertension promotes vascular inflammation, vascular damage, and activated astrocytes and microglia; these events stimulate dysfunctional arterial dilation, the generation of pro-inflammatory stimuli and enhanced levels of reactive oxygen species (ROS) and reactive nitrogen species (RNS; Iadecola et al., 2009). Consequently, cerebral blood flow is significantly reduced and mitochondrial damage becomes pervasive. Ultimately, these physiological cascades manifest in a neuronal energy crisis, neuronal damage, apoptosis, neuro-degeneration, inflammation and finally, AD (Iadecola et al., 2009).

The deleterious structural and functional alterations in cerebral circulation that are associated with hypertension are potentially reversible (Lipsitz et al., 2005), presumably, improving the cerebral blood flow may also lead to improvements in cognitive performance. Li et al. (2011) observed that the treatment of VRF’s such as hypertension reduced the risk of late-onset AD and the progression from MCI to AD; Deschaintre et al. (2009) also reported that the treatment of VRF’s resulted in slower cognitive decline in individuals with AD. Similarly, antihypertensive treatment utilizing pharmacotherapy has been shown to offer protection against dementia in elderly individuals with hypertension (Forette et al., 2002) and to increase the velocity of cerebral blood flow and improve the distensibility of the carotid artery, supporting a correlation between blood pressure reduction and increased cerebral blood flow (Lipsitz et al., 2005).

## Pro-Inflammatory Markers, Systemic Inflammation and Cytokine Responses to AD and Hypertension

Chronic low grade systemic inflammation is a condition characterized by the persistent activation of the bodies intrinsic immune system and is perpetuated by the release of pro-inflammatory cytokines from immune-related cells (Swardfager et al., 2010). Chronic low grade systemic inflammation is believed to contribute to the development and clinical trajectory of conditions such as hypertension and AD (Swardfager et al., 2010). Subsequently, besides the hallmark neuropathology associated with AD chronic low-grade inflammation is also considered a hallmark of AD that may be influencing the neurodegenerative progression of the disease (Wyss-Coray, 2006; Swardfager et al., 2010). Similarly, chronic low-grade inflammation is recognized as a hallmark of hypertension and is attributed, in part, to the etiology of vascular disease (Boos and Lip, 2006; Edwards et al., 2007).

Elevated inflammatory biomarkers that share a clinical association with AD and hypertension include; interleukin (IL) -1β and -6, acute phase C reactant protein (CRP) and tumor necrosis factor (TNF-α; Boos and Lip, 2006; Edwards et al., 2007; Swardfager et al., 2010). Specifically, TNF-α can induce apoptic cell death and inflammation (Swardfager et al., 2010), and is positively correlated with blood pressure levels (Edwards et al., 2007). Dysregulated TNF-α production is implicated in a variety of human diseases including AD (Swardfager et al., 2010) with high levels of this cytokine associated with dementia (Bruunsgaard et al., 1999). IL-6 has been characterized as both a pro- and anti-inflammatory cytokine (Tilg et al., 1997), on one hand IL-6 expression is stimulated by the production of TNF-α and IL-1 (both pro-inflammatory cytokines), on the other hand it is also responsible for suppressing the production of these two inflammatory markers (Petersen and Pedersen, 2005). Furthermore IL-6 is involved in upregulating the expression of anti-inflammatory cytokines IL-10 and IL-1ra (Steensberg et al., 2003).

## Exercise as a Modifiable Risk Factor

Exercise is recommended as a therapeutic intervention for hypertension, atherosclerosis (Cardoso et al., 2010; Cornelissen and Smart, 2013), MCI and AD (Colcombe and Kramer, 2003; Heyn et al., 2004; van Uffelen et al., 2008; Aarsland et al., 2010; Baker et al., 2010; Smith et al., 2010; Sofi et al., 2011; Hess et al., 2014; Groot et al., 2016) and an inverse relationship exists between levels of physical activity and levels of chronic low grade inflammation (Edwards et al., 2007). Specifically, aerobic exercise incites endothelium-dependent vasodilation via the upregulation of NO production, and with regular adherence, inhibits age associated loss in endothelium-dependent vasodilation and restores levels in previously sedentary individuals (DeSouza et al., 2000). Thus, most studies have focused on aerobic exercise due to shear wall stress and the subsequent release of NO. Moreover, prevalent in the broader literature is evidence suggesting that exercise training targeting cardiovascular fitness (VO_2_^peak^) may provide neuroprotective benefits and moderate the structural and functional neuronal changes associated with conditions such as MCI and dementia (Burns et al., 2008; Honea et al., 2009; Bugg and Head, 2011). Evidence derived from animal models indicates that aerobic exercise has the ability to facilitate improvements in angiogenesis, neurogenesis, and learning and memory in rats (Cotman and Berchtold, 2002; Mattson et al., 2002), and in the mouse model, aerobic exercise has been shown to inhibit the evolution of Alzheimer’s-associated neuropathology (Adlard et al., 2005). Current literature suggests that exercise taken up in midlife by healthy adults facilitates improvements in various domains of cognitive functioning and decreases the chances of developing dementia in later life (Laurin et al., 2001; Ahlskog et al., 2011; DeFina et al., 2013). Recent MRI studies have reported a link between brain atrophy and cardiovascular fitness in AD (Burns et al., 2008; Honea et al., 2009), and Erickson et al. (2011) reported increased hippocampal volumes in the brains of healthy individuals who participated in exercise training compared to sedentary controls. A growing number of training studies have investigated the effect of physical activity on the neurocognitive performance outcomes of people at risk of, and living with dementia (Lautenschlager et al., 2008; Brown et al., 2009; Kemoun et al., 2010; Lam et al., 2011; Cheng et al., 2014), subsequently, this literature does offer some support for exercise as a mitigating or stabilizing intervention in relation to some cognitive domains.

Exercise provides an anti-inflammatory environment within the body, post exercise circulating cytokines remaining in the plasma are IL-6, IL-10 and IL-1ra (Petersen and Pedersen, 2005). Exercise increases IL-6 transcription rates (Keller et al., 2001) and during exercise the IL-6 protein is expressed in contracting muscle fibers (Penkowa et al., 2003; Hiscock et al., 2004), markedly increasing circulating levels (Pedersen and Hoffman-Goetz, 2000; Pedersen et al., 2003). Even moderate exercise has been demonstrated to induce marked increases in IL-6 in both the young and elderly. These increases in IL-6 plasma levels are exponential relative to exercise intensity, duration, endurance and recruited muscle mass (Bruunsgaard et al., 1999; Petersen and Pedersen, 2005).

## Remote Limb Ischemia to Affect Distant Organs

The concept that remote ischemic conditioning (RIC) of a limb can support and improve the healthy functioning of distant organs such as the kidneys, heart and the brain has been successfully demonstrated through techniques such as RIC (Hess et al., 2015) and physiological ischemic training (PIT; Ni et al., 2015).

### Remote Ischemic Conditioning

Originally ischemic conditioning (IC) was developed as a cardio protective application (Murry et al., 1986) for patients with cardiovascular arterial disease (CAD) and myocardial ischemia. By stimulating ischemic and hypoxic events via the direct occlusion of a coronary artery, the impact and size of future myocardial infarction was reduced significantly (Przyklenk et al., 1993). Initially IC was an invasive procedure administered by directly occluding coronary arteries for short periods of time, enough to induce small doses of the injurious agents ischemia and hypoxia (Murry et al., 1986). Subsequently, non-invasive applications of IC have been investigated and the benefits of RIC have been illuminated; that is, inducing ischemia in a healthy limb stimulates endogenous protective pathways (Iadecola and Anrather, 2011) that are transferable from one organ to another distant organ (Bøtker et al., 2010; Davies et al., 2013; Sloth et al., 2014). RIC involves the repetitive inflation and deflation of a BP cuff placed around a limb at pressures above SBP (Hess D. C. et al., 2016).

Similar to the heart, the brain can also be conditioned with ischemia and hypoxia (Kitagawa et al., 1990). Recently, RIC has also been demonstrated to stimulate endogenous neuroprotective pathways (Hougaard et al., 2014) and increase cerebral blood flow (Hess D. C. et al., 2016). Mouse models of vascular cognitive impairment (VCI; Bink et al., 2013) have demonstrated that when compared to the control cohort, RIC administered daily for 2 weeks resulted in less inflammation, less β-amyloid deposition, reduced white and gray matter damage, increased cerebral blood flow and improved cognition. Furthermore, RIC has also been implicated in enhancing neuroplasticity, Cherry-Allen et al. (2015) reported significant improvements in motor learning that were not associated with the ischemic trained limb. Although the hypothesized improvements in cognitive learning were not forthcoming in this study, the researchers postulated that this was most likely due to the difficult nature and the narrow assessment framework of the cognitive assessment task that was utilized and not necessarily indicative that cognitive improvements did not occur. Consequently, to elucidate the cognitive benefits of remotely induced ischemia and hypoxia the authors recommended that future research in this domain utilize cognitive assessment tasks that assess broad ranges of neural regions and networks.

Whilst the mechanisms involved in the remote signaling and in the stimulation of endogenous pathways to facilitate protective responses and structural changes in distant organs are not fully understood, evidence obtained through animal models and clinical trials supports a number of mechanisms involving; blood borne factors (Shimizu et al., 2009; Koch, 2010; Koch et al., 2014; Hess D. C. et al., 2016) induced by peripheral nerves (Jensen et al., 2012; Redington et al., 2012), epigenetic modulations of the genome (Stowell et al., 2010; Brand and Ratan, 2013; Thompson et al., 2015), and immune and anti-inflammatory responses (Konstantinov et al., 2004). Consequently, it is the interaction of blood borne and neuronal factors that are postulated to both initiate and transmit these signals to the brain.

### Physiological Ischemic Training

Inspired by RIC, recently the feasibility of PIT to stimulate remote ischemia has also been investigated. PIT is a technique whereby skeletal muscle is subjected to intense contraction via isometric contraction or tourniquet in order to stimulate physiological ischemia (Ni et al., 2015). In animal models, PIT applied eight times daily for 4 weeks to a normal healthy limb has been shown to upregulate vascular endothelial growth factor (VEGF) and facilitate angiogenesis improving blood flow and capillary supply in a remote pathological ischemeic limb (Shen et al., 2009; Zhao et al., 2011). In clinical trials, PIT using isometric handgrip exercise performed at 50% maximal voluntary contraction (MVC) by patients with coronary artery disease and a coronary artery occlusion significantly increased collateral blood flow in the myocardium (Lin et al., 2012). The proposed mechanisms responsible for the effects of PIT include; upregulation in circulating VEGF and VEGF mRNA, angiogenesis (Liu et al., 2004), the differential expression of proteins involved in cell migration and energy metabolism (Gao et al., 2011) and increased systemic endothelial progenitor cells (EPCs; Wan et al., 2011). Unlike RIC, the efficacy of PIT to neural applications has not yet been investigated.

Most encouraging is the potential neuroprotective implications that ischemic training may offer those with MCI, AD and VaD. Whilst the protocols between these two techniques differ from each other and the extent of commonality of the signaling and protective mechanisms involved is still the subject of investigation, both of these techniques involve the activation of endogenous signaling and protective pathways and, according to recent literature, also appear to engage some shared mechanisms. Encouragingly RIC administered to patients aged 80–95 years old with intracranial atherosclerosis stenosis was found to be both safe and effective in stroke prevention and treatment (Meng et al., 2015). Moreover, the principles that support the efficacy of RIC and PIT also support the feasibility of a hypothesis that IET performed by elderly individuals might promote healthy neural functioning and boost cognitive performance.

## Isometric Exercise Training

Traditionally, aerobic endurance training has been the preferred type of physical activity recommended for blood pressure management, however, current thinking does vary with respect to this. IET involves a single sustained muscle contraction against an immovable load or resistance with no, or minimal, change in length of the involved muscle group. An increasing body of evidence suggests that IET promotes significant reductions in resting systolic and DBPs in hypertensive and normotensive men and women (Devereux et al., 2010; Wiles et al., 2010; Badrov et al., 2013a,b; Millar et al., 2013). Previously, isometric exercise has been associated with exaggerated hypertensive responses, however, data from recent analyses suggests that isometric resistance exercise may elicit blood pressure reductions greater than those seen with dynamic aerobic and resistance exercise (Cornelissen and Smart, 2013) and has been safely implemented among a cohort of hypertensive elderly women, 70–82 years old (Olher Rdos et al., 2013). Specifically, acute isometric hand grip training (IHG) has been shown to improve resting endothelium-dependant vasodilation (McGowan et al., 2006). Recent meta-analyses have reported IET to elicit greater reductions in resting SBP than those observed in dynamic resistance training, dynamic aerobic exercise training, and training consisting of both dynamic resistance and aerobic activity (Cornelissen and Smart, 2013; Carlson et al., 2014). The magnitude of effect is comparable to that of monotherapy with beta-blockade (Wong and Wright, 2014).

The precise mechanism(s) of the anti-hypertensive effect(s) of isometric exercise remain unclear, however, with blood borne factors postulated as one of the mechanism involved in the remote signaling and stimulation of endogenous protective pathways in RIC techniques (Shimizu et al., 2009; Koch, 2010; Koch et al., 2014; Hess D. C. et al., 2016), it could be the case that for IET the initial stimulus is repeated exposure to transient increases in blood flow (reactive hyperemia) that result after short periods of ischemia. Edwards et al. (2007) suggest that reduced peripheral vascular resistance facilitated via neurohormonal and structural adaptations might also explain the antihypertensive effects of exercise. The reactive hyperemia elicited during a 2 min IHG training may occur due to either partial or full occlusion of the brachial artery. Previous research suggests that full occlusion of blood flow occurs at approximately 55%–75% of MVC with higher occlusion thresholds evident in individuals who were able to exert a greater handgrip force (Barnes, 1980). The production of a number of metabolites such as beta endorphins, prostaglandins and hypoxia-inducible factor-1 α (HIF 1 α) are postulated to result from ischemia induced by full or partial restriction of the brachial artery during IHG exercises (Stiller-Moldovan et al., 2012; Wong and Wright, 2014; Wong et al., 2015). These metabolites play a number of roles including, supporting the immune system, managing inflammation (Cabot et al., 1997; Pedersen and Hoffman-Goetz, 2000), vasodilation and vasoconstriction (Davidge, 2001), stimulating angiogenesis, and tissue repair and regeneration (Wang et al., 2014). It may be that the intensity of the hand grip contraction employed during IHG exercise determines the existence or absence of reactive hyperemia; intuitively, even at intensities less than 55% of MVC partial occlusion of blood flow is likely. Indeed, previous work with isometric exercise suggests that intensities as little as 10%–14% of MVC may be sufficient to elicit partial occlusion to blood flow, inducing ischemia and the resulting metabolic production that might be contributing to BP reductions (Wiles et al., 2010; Baross et al., 2012; Hess N. C. et al., 2016). Utilizing lower intensity IHG exercise for BP reduction may prove beneficial in the design of exercise programs for the frail and elderly, elderly people may struggle to complete IHG exercise at 30% MVC yet may still benefit from an isometric exercise program at 10% MVC. Considering the anti-hypertensive effects elicited by isomeric exercise and the purported mechanisms responsible for this, it is also plausible that IET may play an effective role in the management of hypertension at the MCI stage of AD and in conjunction prove to be a significant strategy in the prevention, attenuation or delay of progression to AD. Further research is probably warranted in this area.

## Conclusion

The contribution of cerebral hypoperfusion to the development of MCI and AD is receiving increasing attention. A review of the current literature supports both a strong relationship between the contribution of cerebral hypoperfusion to the development of MCI and sporadic AD and a strong link between untreated hypertension and neurodegenerative processes. In conjunction with the therapeutic benefits elicited via the application of RIC and PIT, evidence suggesting that IET may be more efficacious at inducing anti-hypertensive responses than aerobic exercise and resistance training provokes questions relating to the possible role that IET might play in facilitating reparations to the vasculature, increasing cerebral blood flow, reducing chronic low grade inflammation and possibly improving cognitive performance.

Future IET research protocols should seek to extend investigations beyond their traditional hypertensive enquiry to explore the effects of isometric exercise on cognitive performance outcomes. These studies should seek to incorporate diverse arrays of subgroups; possible subgroups might include groups that are gender specific; groups that constrain age ranges to within ±5 years as this may help to elucidate the effects of age on IET protocols, and groups where the characterization of pre-dementia syndromes such as MCI and dementia are uniform across the population sample as different dementias are likely to respond differently to the same treatment. Incorporating techniques such as magnetic resonance imaging, blood analysis and genotype profiling would further assist in our understanding of the mechanisms of ischemic training on the brain, BP and cognition.

## Author Contributions

NCLH formulated the idea for the review, contributed to the creation of the figures and wrote the first draft of the manuscript. NAS provided critical and significant feedback as to content and structure throughout the writing process. A process of critical revision by NAS and subsequent editing by NCLH was undertaken until both authors were able to agree on the version to be published. Both authors contributed to and have approved the final manuscript.

## Conflict of Interest Statement

The authors declare that the research was conducted in the absence of any commercial or financial relationships that could be construed as a potential conflict of interest.
